# Neurocysticercosis With Collision Metastasis of Ovarian Serous Adenocarcinoma: A Case Report

**DOI:** 10.7759/cureus.82992

**Published:** 2025-04-25

**Authors:** Adam Bowen, Jason Grullon, Hanish Polavarapu, John Morrison

**Affiliations:** 1 Internal Medicine, Mclaren Greater Lansing Hospital, Lansing, USA; 2 Neurosurgery, Upstate University Hospital, Syracuse, USA

**Keywords:** collision-metastasis, epilepsy, neurocysticercosis, serous ovarian adenocarcinoma, taenia solium

## Abstract

Neurocysticercosis (NC), a parasitic infection caused by *Taenia solium*, is an increasingly recognized cause of seizures in the United States, particularly among individuals from endemic regions. This report describes a diagnostically challenging case of a 51-year-old Vietnamese woman who presented with new-onset seizures and was initially treated for NC following craniotomy. Despite antiparasitic therapy, persistent symptoms and progressive imaging changes prompted further intervention, ultimately revealing a metastatic ovarian serous adenocarcinoma adjacent to the prior NC lesion. This rare instance of collision metastases, with an uncommon instance of ovarian cancer metastasizing to active NC, underscores the complexity of differentiating NC from neoplastic disease, emphasizing the need for a broad differential in patients with intracranial lesions and risk factors for both infectious and malignant processes.

## Introduction

Neurocysticercosis (NC) is a parasitic disease caused by *Taenia solium* larvae infecting the central nervous system. Although most commonly found in developing countries, NC has a prevalence of 0.2-0.6 per 100,000 people in the United States and causes 50,000 deaths annually. Latin America, Asia, and sub-Saharan Africa are particularly affected, with the World Health Organization predicting anywhere from two to eight million cases a year [1].

Clinical manifestations of NC include seizures, intracranial hypertension, focal neurologic deficits, and psychiatric symptoms. NC has been associated with various malignancies, although the nature of this relationship remains unclear. In an autopsy-based study of 2,012 cases, Cavellani et al. found that all patients with concurrent neoplasia and cysticercosis (0.4% of the sample) had malignant tumors - most commonly of the digestive system (62.5%), followed by the respiratory (25%) and gynecological systems (12.5%) [1].

The oncogenic effects of NC have been suggested by various pathogenic mechanisms, including modulation of the immune response, transfer of genetic material to the host resulting in DNA damage, chronic inflammation, and inhibition of tumor suppressor genes [2].

While metastases to the brain commonly originate from primary cancers like lung, breast, and melanoma, ovarian metastasis is rare, occurring in around 2%-3% of patients [3,4]. The radiologic features of NC are quite similar to those of brain abscess, meningioma, and brain metastasis, increasing the importance of screening for patients coming from endemic regions [5].

This case highlights the importance of maintaining a broad differential diagnosis in patients presenting with new-onset seizures, particularly in individuals from endemic regions or with a history of malignancy.

## Case presentation

A 51-year-old Vietnamese female presented to the hospital in a postictal state with new-onset seizure and stiffness in her right upper and lower extremities. Communication was challenging, as the patient was communicating through her son and daughter with translation. The patient experienced full-body stiffening, convulsions, tongue biting, and confusion upon being taken to the hospital. Upon further questioning, it was revealed that she had undergone a left breast lumpectomy with sentinel lymph node biopsy five years ago due to stage 1 invasive ductal carcinoma. There was no evidence of lymph node spread or metastases, and she was treated post-surgically with anastrozole. She had also had uterine cancer, which was surgically resected via total abdominal hysterectomy with bilateral salpingo-oophorectomy the year prior to her breast surgery.

**Figure 1:**
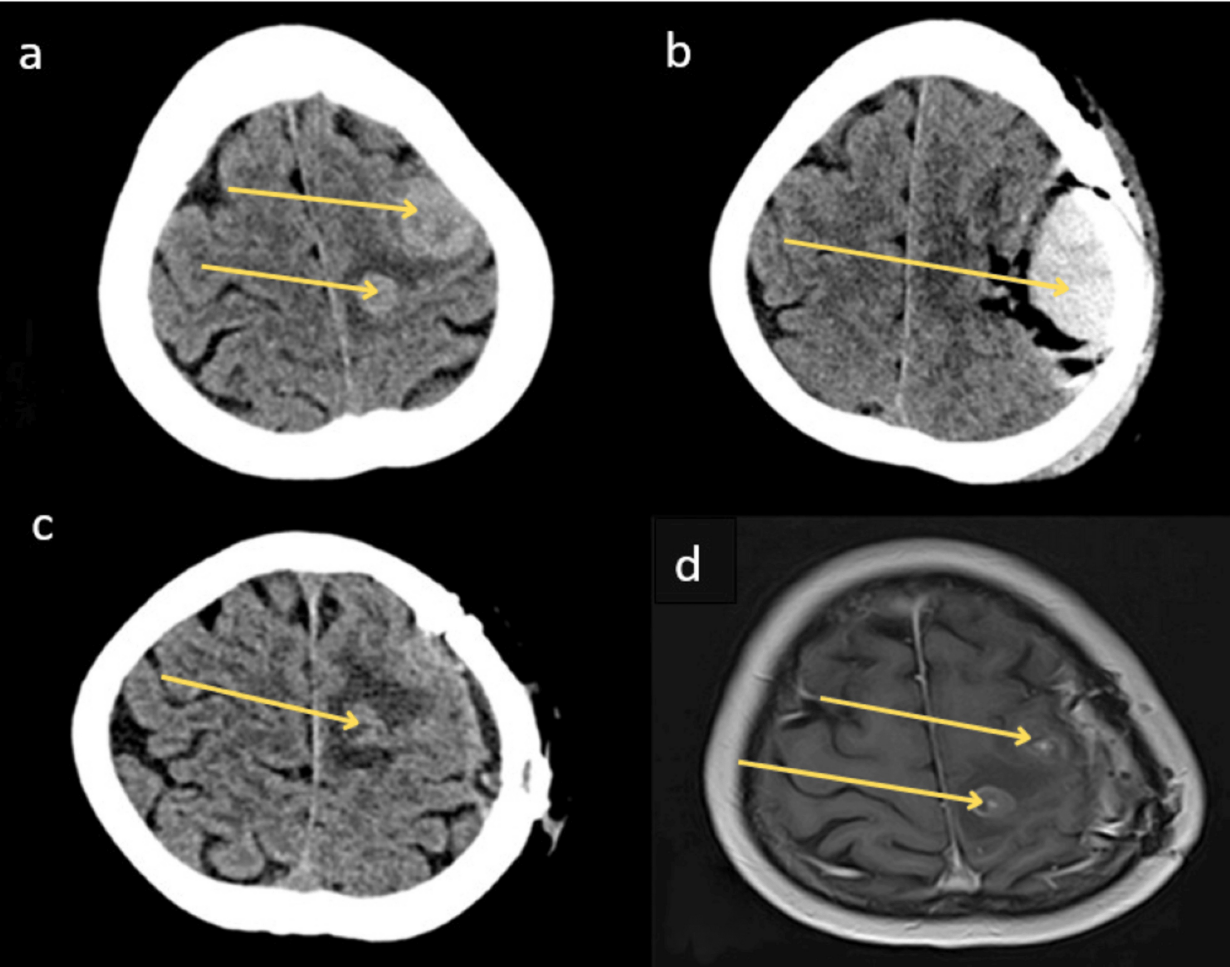
Timeline of Neuroimaging Findings in a Patient With Neurocysticercosis and Concomitant Metastatic Disease (a) Non-contrast head computed tomography (NCHCT) obtained during the patient’s initial emergency department visit shows two hyperattenuating parenchymal lesions within the left frontoparietal lobule (arrows), initially suspected to represent metastatic disease. (b) Post-operative NCHCT, one day after the initial craniotomy, shows resection of the intra-axial lateral lesion (arrow) with a 2.2 cm extra-axial hematoma beneath the skull flap. The medial lesion remains unchanged. (c) NCHCT, approximately two months post-op during evaluation for recurrent seizure, reveals persistent left frontal vasogenic edema (arrow) with a 1.1 cm nodular density, initially presumed to be a retained intracranial abscess. (d) T1-weighted magnetic resonance imaging (MRI), performed approximately nine months after the initial presentation, demonstrates a slight enlargement of the left medial frontal convexity (arrows) enhancing lesion with a mild increase in vasogenic edema. The left lateral lesion remains stable.

An initial non-contrast head computed tomography (NCHCT) demonstrated two left hyperattenuating frontoparietal lesions in the gray-white junction, raising a reasonable assumption of metastases (Figure 1a). However, a CT scan of the chest, abdomen, and pelvis on the same day did not corroborate metastases. Due to the need for definitive diagnosis and lesion accessibility, a craniotomy was performed via a left posterior frontal approach with stealth guidance. Purulence was observed during the procedure, and samples were taken for culture and pathology. The multifocal abscess was thoroughly drained and irrigated prior to closure. The initial diagnosis of NC was based on intraoperative findings of purulence, radiographic features consistent with vesicular NC, and confirmatory histopathology from the first craniotomy, which identified parasitic structures. Cultures were negative for bacteria, and there was no evidence of malignancy on histopathology or cytology at that time.

Post-operative NCHCT demonstrated post-operative changes and a medial remnant (Figure 1b). The patient experienced right-sided hemiparesis post-operatively, suggestive that the resolving hematoma was likely the cause. A diagnosis of NC was confirmed 12 days after the craniotomy, and the patient was prescribed albendazole 15 mg/kg/day, divided into two doses, and praziquantel at 50 mg/kg/day, divided into three doses, consistent with standard antiparasitic regimens for parenchymal NC. To manage perilesional edema, corticosteroid therapy was transitioned from intravenous dexamethasone dosed at 4 mg every six hours to an oral prednisone taper, starting at approximately 60 mg daily, with a gradual reduction over two weeks. For seizure prophylaxis, the patient was started on levetiracetam (Keppra), initially loaded with a 1,000 mg intravenous bolus, followed by a maintenance dose of 500 mg orally twice daily.

The patient was advised to undergo two weeks of treatment and have repeat magnetic resonance imaging (MRI) scans of the brain to monitor the resolution of the presumed residual NC. The patient had many repeated emergency department (ED) visits following the craniotomy and standard of care (SOC) NC treatment. The patient had two ED visits for leukopenia and witnessed seizure. A repeat NCHCT during the visit demonstrated persistent vasogenic edema and presumed retained intracranial abscess (Figure 1c). An MRI revealed two lesions, with edema surrounding the smaller lesion, and the patient was restarted on dexamethasone for associated vasogenic edema (Figure 1d). Two months later, the patient experienced another seizure and was evaluated with a lumbar puncture and empiric treatment for NC, which she tolerated well. However, CSF culture and cysticercosis antibody immunoglobulin G (IgG) ELISA (Enzyme-Linked Immunosorbent Assay) were negative. At this point, the assumption was that the NC was not responding to anti-parasitic management, as clearance can sometimes be protracted.

**Figure 2:**
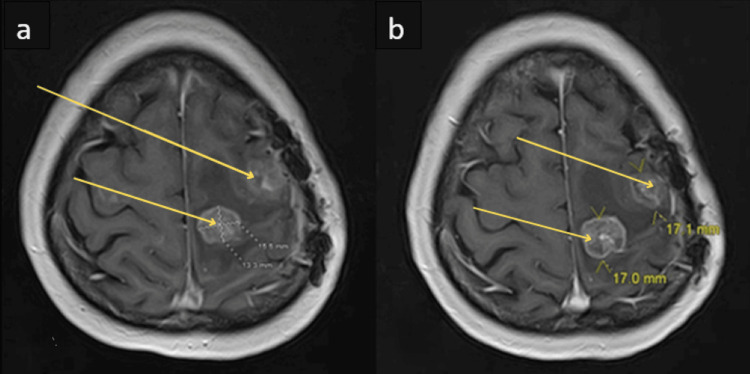
Progressive Enlargement of the Medial Frontal Lesion on Serial Magnetic Resonance Imaging (a) T1-weighted magnetic resonance imaging (MRI), performed approximately 11 months after the initial craniotomy, showing worsening medial edema, with the lesion now measuring 13.3 × 15.5 mm (arrows). (b) Follow-up T1-weighted MRI, performed approximately 13 months post-craniotomy, demonstrating a further increase in vasogenic edema, with the medial lesion enlarged to 17 mm (arrows), despite prior antiparasitic and corticosteroid therapy.

Her seizures continued despite medication compliance, including an uptitration of levetiracetam to the maximum recommended outpatient dose of 1,500 mg twice daily, and she was continued on steroids for perilesional edema. Nine months after the initial craniotomy, the steroids were discontinued, and a repeat MRI was performed, revealing progression and growth in the size of the left medial frontal lesion with worsening edema (Figure 2a). Two months later, the patient visited the ED with repeated seizures. It was presumed that a third course of albendazole and praziquantel could be beneficial for the edema and medical resolution. Despite the lack of radiologic response to previous antiparasitic therapy, the working diagnosis remained refractory NC, due to the patient’s known history and persistent imaging features.

In the final admission, the patient had gone to the ED with continued seizures and imaging consistent with an increased lesion size, without improvement from the third antiparasitic treatment (Figure 2b). Despite the risk of exacerbating her right hemiparesis, a craniotomy was performed to remove the suspected medication-resistant NC. Intraoperatively, an encapsulated tumor consistent with metastatic carcinoma was found. The final pathology revealed moderate to poorly differentiated metastatic ovarian serous adenocarcinoma. A final CT chest and abdomen showed a 1.2 x 0.9 cm density near the left adnexal clips of the ovary, as the presumed origin of the metastases. 

## Discussion

Parasitic diseases are known to increase cancer risk. A case-control study supported that NC is a risk factor for developing cerebral gliomas [3]. The study suggested a spatial relationship between calcification and gliomas, with infective foci surrounding and adjacent to the primary gliomas in most cases. In contrast, our patient had adjacent metastases rather than a primary neoplasm, and imaging did not demonstrate calcifications. Multiple studies have demonstrated a higher incidence of malignant neoplasms with NC, including hematologic cancers, gliomas, and other solid cancers [1,6]. To the best of our knowledge, our report is the first report of “collision metastasis,” where serous ovarian adenocarcinoma migrated adjacent to an NC infection focus.

The initial diagnosis of NC in our case was based on intraoperative visualization of purulence, histopathologic confirmation of parasitic structures consistent with *T. solium*, and radiographic features congruent with the vesicular stage of NC. Cultures were negative for bacterial infection, and cytopathology revealed no evidence of malignancy at that time. Cerebrospinal fluid (CSF) serology and cytology, conducted later in the disease course, were non-diagnostic, underscoring the importance of early tissue-based confirmation.

Some argue that NC should be considered in the differential diagnosis of potential brain metastases in developing countries. Cacho-Díaz et al. presented 18 patients with NC and concurrent metastases, suggesting a possible association between the two [7]. However, none of their presented patients had metastases to the brain. Our patient’s case was particularly rare, initially diagnosed and treated for presumed metastasis, but later discovered to have NC. The patient was then diagnosed with a concomitant metastasis that was present during the initial surgery. This is especially instructive, as it shows that oncogenesis should always be on the differential for NC.

NC has a biphasic course, starting with a vesicular phase and then progressing to a colloidal phase, with significant local inflammation caused by larval degeneration [8]. This inflammation conversion in previous studies is thought to be due to epigenetic modulation of microglial cells. Microglia in NC have been observed to suppress M1-inflammatory activation. This may decrease the likelihood of glioma development or metastasis to the brain but increase metastases elsewhere in the body. The temporality in our patient suggests possible metastasis that was enabled in the initial vesicular NC stages, which was then predisposed to the brain during the NC inflammatory transition.

## Conclusions

This case report presents a rare and diagnostically challenging instance of collision brain metastases occurring alongside symptomatic NC. This case exemplifies a complex and evolving diagnostic process. The initial diagnosis of NC was based on intraoperative purulence, radiographic features consistent with vesicular NC, and confirmatory histopathology demonstrating parasitic structures. The subsequent diagnosis of metastatic carcinoma was made following a second craniotomy due to radiographic progression, with pathology revealing no parasitic elements but confirming metastatic ovarian adenocarcinoma. Collision metastasis is exceedingly rare, with NC potentially serving as a nidus for uncommon ovarian brain metastasis. This case further reinforces the potential association between NC and metastatic disease, highlighting the need for continued investigation into their oncogenic interplay. The temporal progression of metastases and corresponding imaging findings provides valuable insights into the proposed biphasic NC oncogenic model. Additionally, this case underscores the importance of expanding public health resources to better address the rising incidence of NC in underserved populations across the United States.
